# Down-regulation of the transcription factor snail in the placentas of patients with preeclampsia and in a rat model of preeclampsia

**DOI:** 10.1186/1477-7827-10-15

**Published:** 2012-02-23

**Authors:** Larisa Fedorova, Cara Gatto-Weis, Sleiman Smaili, Nauman Khurshid, Joseph I Shapiro, Deepak Malhotra, Terrence Horrigan

**Affiliations:** 1 Department of Medicine, University of Toledo School of Medicine, Toledo, OH 43614, USA; 2 Department of Pathology, University of Toledo School of Medicine, Toledo, OH 43614, USA; 3 Department of Obstetrics and Gynecology, University of Toledo School of Medicine, Toledo, OH 43614, USA

**Keywords:** Preeclampsia, Placenta, Snail, Trophoblast, E-cadherin

## Abstract

**Background:**

Placental malfunction in preeclampsia is believed to be a consequence of aberrant differentiation of trophoblast lineages and changes in utero-placental oxygenation. The transcription factor Snail, a master regulator molecule of epithelial-mesenchymal transition in embryonic development and in cancer, is shown to be involved in trophoblast differentiation as well. Moreover, Snail can be controlled by oxidative stress and hypoxia. Therefore, we examined the expression of Snail and its downstream target, e-cadherin, in human normal term, preterm and preeclamptic placentas, and in pregnant rats that developed preeclampsia-like symptoms in the response to a 20-fold increase in sodium intake.

**Methods:**

Western blotting analysis was used for comparative expression of Snail and e- cadherin in total protein extracts. Placental cells expressing Snail and e-cadherin were identified by immunohistochemical double-labeling technique.

**Results:**

The levels of Snail protein were decreased in human preeclamptic placentas by 30% (*p < 0.01) *compared to normal term, and in the rat model by 40% (*p < 0.001) *compared to control placentas. In preterm placentas, the levels of Snail expression varied, yet there was a strong trend toward statistical significance between preterm and preeclamptic placentas. In humans, e-cadherin protein level was 30% higher in preeclamptic *(p < 0.05) *placentas and similarly, but not significantly *(p = 0.1)*, high in the preterm placentas compared to normal term. In the rat model of preeclampsia, e-cadherin was increased by 60% (*p < 0.01)*. Immunohistochemical examination of human placentas demonstrated Snail-positive staining in the nuclei of the villous trophoblasts and mesenchymal cells and in the invasive trophoblasts of the decidua. In the rat placenta, the majority of Snail positive cells were spongiotrophoblasts of the junctional zone, while in the labyrinth, Snail-positive sinusoidal giant trophoblasts cells were found in some focal areas located close to the junctional zone.

**Conclusion:**

We demonstrated that human preeclampsia and the salt-induced rat model of preeclampsia are associated with the reduced levels of Snail protein in placenta. Down-regulation of the transcription factor Snail in placental progenitor cell lineages, either by intrinsic defects and/or by extrinsic and maternal factors, may affect normal placenta development and function and thus contribute to the pathology of preeclampsia.

## Background

Preeclampsia, a devastating, life-threatening, human pregnancy complication, develops in 3-10% of pregnancies. Clinical symptoms include a sudden onset of hypertension accompanied by proteinuria, edema and often fetal growth restriction [1]. The placenta of patients with preeclampsia is malformed [2]. It has been hypothesized that abnormalities in trophoblast function may contribute to placental defects associated with preeclampsia [3]. An increased proliferation of progenitor villous cytotrophoblastic cells (VCT) and an augmented apoptosis of the terminally differentiated syncytiotrophoblast (SynTB) results in thinning and distortion of the syncytial layer, and in the appearance of multinucleated syncytial buds [2,4,5]. The villous injury in preeclampsia is associated with poor perfusion/oxygenation of the intervillous space, utero-placental hypoxia, and oxidative stress [6,7]. Proper oxygenation of the placenta is achieved through remodeling of the maternal spiral arteries by a population of VCTs which, upon contact with the decidua, acquire an invasive phenotype and are hence called extravillous trophoblast (EVT). In preeclampsia, EVT invasion is limited and vascular transformation is incomplete [2]. Transformation of polarized, non-motile, proliferative epithelia-like VCT into highly invasive EVT requires loss of polarity, down-regulation of epithelia-specific adhesion molecules, up-regulation of extracellular matrix receptors, and de novo expression of matrix degradation proteins [8]. This transformation to some extent resembles the process of epithelial-mesenchymal transition (EMT), which is common in embryonic development and in metastastic cancers [9]. EMT is commonly initiated by down-regulation of the adhesive junction protein, e-cadherin. Similarly, in normal placenta, invasive and migratory, individual and aggregated EVTs exhibit reduced, discontinuous expression of e-cadherin [10]. Conversely, in preeclampsia, EVTs fail to down-regulate e-cadherin during the initiation of invasion in anchoring cell columns, and also during the fusion of the giant multinucleated cells in the inner myometrium [11-13].

In embryogenesis and cancer, e-cadherin expression is under the control of the Wnt pathways and/or the zinc finger transcription factor Snail [9,14]. At the transcriptional level, Snail is regulated by many signaling pathways including those that are crucial for placental development, such as RAF/MEK/ERK and PI3K/AKT/mTOR [15]. Post-transcriptional regulation of the highly unstable Snail protein is under control of GSK-3β, a member of WNT signaling cascades, and hypoxia-inducible factor alpha (HIF-1α) [16,17]. Moreover, Snail expression is also sensitive to hypoxia, either directly through activation of the HIF-1α responsive element on its promoter, or by such mediators of hypoxic signals as EGF, TGFα,βs and TNFα [14]. Snail up-regulation by HIF-1α in cultured human first trimester villous explants reduces e-cadherin expression and activates an invasion program [18]. Also, Snail has been shown to down regulate e-cadherin during syncytialization of BeWo trophoblast cell line [19]. In cultured rodent trophoblast cells, Snail expression correlates with the transition from proliferating to terminally differentiated stages [20,21]. It also has been shown that the number of Snail positive EVTs in the basal plate of the human placenta is increased in preeclampsia and HELLP syndrome compared to normal [22], which does not concur with a commonly accepted notion of limited invasive capacity of the EVT in preeclampsia.

Disturbed placenta is believed to be a primary cause of excessive maternal inflammatory response, which in turn instigates endothelial dysfunction and the increased vascular reactivity in preeclampsia [1]. Defects in endothelial function contribute to derangements of sodium-volume homeostasis and thus compromise cardiovascular adaptations to pregnancy. A variety of animal models which, to a different degree, mirror the vascular pathology seen in human preeclampsia, have been developed [23]. Several studies demonstrated that high sodium uptake during the last week of the rat pregnancy can induce such manifestations of preeclampsia as elevated blood pressure, decreased activity of the renin-angiotensin-aldosterone system, renal dysfunction and fetal growth restriction [24-26]. During the last week of normal rat pregnancy, the trophoblast cells invade the mesometrial triangle, thus stimulating vascular remodeling of maternal arteries through replacement of the maternal endothelium [27]. During the same period, maternal blood pressure gradually falls in spite of blood volume expansion and activation of the renin-angiotensin-aldosterone system [26].

The aim of this study was to examine the protein levels of transcription factor Snail and e-cadherin in placental samples from women with preeclampsia, and from normotensive gestationally-matched preterm and term controls at delivery. We also examined Snail/e-cadherin expression in near-term placentas from rats developing preeclampsia-like symptoms in response to high sodium intake.

## Methods

### Collection of placental tissues

The human placenta samples were collected at The Toledo Hospital and St. Vincent Mercy Medical Center in Toledo, Ohio, according to approval of the University of Toledo Biomedical Research Review Board, and after informed patient consent. Placental tissues were collected from 9 patients after normal term delivery, from 8 patients with preeclampsia and from 5 patients with preterm labor. Pregnancies were diagnosed as preeclamptic according to recommendations given by the American Society of Hypertension [28]. All studied preeclamptic placentas were from women who developed severe hypertension (systolic blood pressure ≥ 160, diastolic ≥ 110), and heavy proteinuria (≥ 4 g of protein in a 24 hour urine collection, or ≥ 3+ protein on dipstick measurement on two or more occasions). All 5 preterm deliveries were associated with premature rupture of amniotic membranes. All patients who had normal or premature deliveries did not have any prior diagnosed pregnancy complications. Immediately after delivery, the peripheral parts of placenta, without any visible infarcts or retroplacental clots, were snap frozen in liquid nitrogen or preserved in 10% formalin.

The salt-induced model of rat preeclampsia was described in detail in several publications [24-26]. Ethical protocol for the rat studies was approved by the Animal Care and Use Committee of the Intramural Research Program, National Institute on Aging [24,29]. Briefly, in the experimental group of Sprague-Dawley pregnant rats, regular drinking water was replaced with water containing 1.8% NaCl. The salt overdose regime was started at 13dpc because the last week of normal rat pregnancy is accompanied by blood volume expansion, activation of the renin-angiotensin-aldosterone system and a significant fall in arterial blood pressure. The rat placentas used in the study were extracted from exactly the same control and experimental animal groups (5 and 9 rats respectively) that were thoroughly described earlier [24,29]. After extraction, the whole placentas were snap frozen and sent to our laboratory on dry ice.

### Protein extraction

Large, randomly chosen pieces of human placental tissues (up to 20 g) taken from two areas containing both fetal and uterine surfaces, and entire rat placentas were homogenized in liquid nitrogen. The resulting powder was transferred into ice-cold RadioImmunoPrecipitation Assay (RIPA) buffer, containing 50 mM Tris-HCl, pH 7.5, 150 mM NaCl, 1% Nonidet P-40, 5% sodium deoxycholate, 0.1% SDS, and protease inhibitors (Protease Inhibitor Cocktail, Sigma) and processed as previously described [30].

### Western blotting and immunohistochemistry

Western blotting analysis was carried out using standard protocol as previously described [30]. Briefly, from 10 to 20 μg of protein (equal amounts for each gel) were loaded into each well. Proteins were transferred by semi-dry method and then processed exactly as thoroughly described earlier [30]. ImageJ software based analysis (http://rsbweb.nih.gov/ij) was performed to quantify the bands obtained via Western blot analysis. The area under the curve (AUC) of the specific signal was corrected for the AUC of the actin loading control. The average value for the samples from normal term (human) or control (rat) placentas was set as 1 and other conditions were recalculated correspondingly to allow ratio comparisons.

For double immunohistochemistry of the human placentas, deparaffinized and re-hydrated 5 μm thick placental sections were washed in TBS, blocked in TBS containing 10% goat serum and 5% BSA for 2 hours at room temperature, and probed with anti-Snail antibodies (ab17732, or ab85935, Abcam, Cambridge, MA, USA) diluted 1:200 in 5% goat serum/2.5% BSA in TBS for 1 hour at room temperature and overnight at 4°C. After washing, the slides were boiled in acetic buffer (Vector Laboratories, Burlingame, CA, USA) in a conventional microwave. Then, the slides were probed with anti-e-cadherin antibodies (BD Bioscience, Sparks, MD, USA) diluted 1:150 in 1.5% goat serum in TBS. The endogenous peroxidase activity was blocked with 3% H_2_O_2 _in methanol. E-cadherin antibodies were detected first using anti-mouse HRP-conjugated polymer (Zymed, San-Francisco, CA, USA) for 30 min followed by the color development with DAB. To detect the anti-Snail antibody, we used anti-rabbit ABC-AP kit and Fast Red chromophore according to standard procedure (Vector Laboratories).

Since whole placentas from experimental rats were utilized for protein extraction, we used commercially available slides of definitive rat placenta for immunohistochemical detection of Snail protein (ab4623, Abcam). The slides were boiled in acetic buffer, and then sections were blocked with 1.5% goat serum in TBS for 1 hour at room temperature. The tissue sections were first probed with anti-Snail antibody diluted 1:50 in 1.5% goat serum for 1 hour at room temperature. Afterward, the staining protocol was the same as described for human placenta. Anti-Snail antibodies (ab17732, or ab85935, Abcam) were raised against the same peptide sequence. We tested their specificities by applying the anti-Snail antibodies with this peptide (ab19126, Abcam).

### Statistical analysis

Data are presented as the mean ± standard error of the mean unless otherwise specified. One-way ANOVA followed by Tukey HSD test was used to compare three groups of human samples. Unpaired Student's t-test was used to evaluate the difference between rat groups. Statistical significance was reported at the *p < 0.05, **p < 0.01 and ***p < 0.001 levels.

## Results

The three groups studied were not significantly different from one another with respect to maternal age at time of delivery (Table 1). Average gestational age was also not significantly different between the preterm and preeclampsia groups of patients. Thus, the placenta samples from preterm deliveries served as gestation-matched controls for analysis of Snail/e-cadherin proteins in preeclampsia. The preeclampsia group was heterogeneous with regard to gestational age and fetal birth weight at delivery, yet all samples from preeclamptic placentas were obtained in the preterm period.

**Table 1:** Clinical characteristics of patients in the study

	Normal term deliveries	Preterm deliveries	Preeclampsia
*Patients*	9	5	8
*Maternal age (years)*	29.7 ± 6.4 (22-34)	32.6 ± 7.2 (29-38)	25.8 ± 6.6 (20-36)
*Gestational age at* *deliveries (weeks)*	38.3*± *0.5 (38-39)	35.4 ± 1.1 (34-36)***	31.3 ± 3.1 (28-36)***
*Birth weight (percentile)*	77.3 ± 8.2 (70-90)	65.0 ± 26.9 (25-90)	39.5 ± 34.9 (5-95)*

Values represent group mean ± SD. Numbers in the parenthesis show the range of the values

*p < 0.05 preeclampsia versus both preterm and term deliveries

***p < 0.001 preterm and preeclampsia deliveries versus normal term

**Figure 1:**
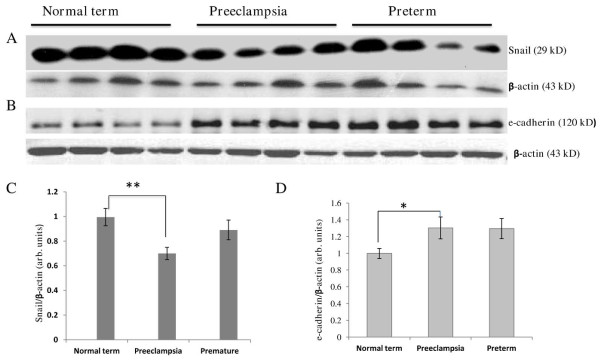
**Western blotting analysis of Snail and e-cadherin expression in the total protein extracts from human placentas**. A representative immunoblot is shown (Snail) from at least four separate runs of each sample (A), and graph (C) demonstrating densitometry analysis of Snail protein levels in normal term, preeclamptic, and preterm placentas (n = 36, 32, 30 respectively). For Snail, the ANOVA test revealed a significant difference among the three groups (p value = 0.0100). However, the Tukey HSD post-hoc test only indicated a statistically significant difference when comparing the normal term group and the preeclampsia group (p value = 0.0085). The difference between the preterm and the preeclamptic samples was not significant (*p = 0.0941*). Representative immunoblot (e-cadherin) from three separate gels (B), and graph showing densitometry analysis of e-cadherin levels in the three groups studied (D) (n = 27, 24 and 15 for normal term, preeclamptic, and preterm placentas respectively). For e-cadherin, the ANOVA test also revealed a significant difference among the three groups (p value = 0.0265). Again, the Tukey HSD post-hoc test only indicated a statistically significant difference when comparing the normal term group and the preeclampsia group (p value = 0.0397). The difference between the preterm and normal term group was not statistically significant (p = 0.1). * p < 0.05; ** p < 0.01

**Figure 2:**
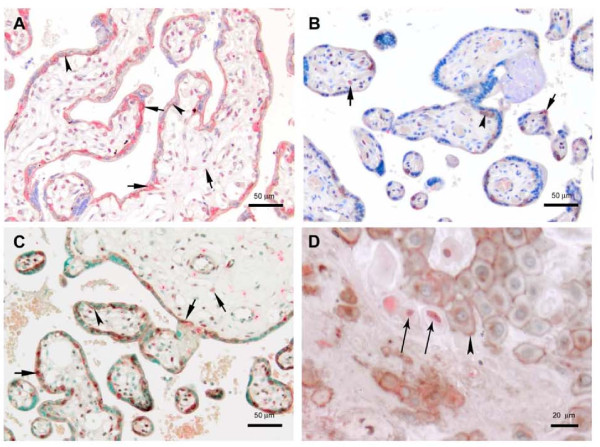
**Immunohistochemical analysis of Snail and e-cadherin proteins expression in human normal term, preeclamptic and preterm placentas**. E-cadherin (stained brown) is present in the basement membranes of the villi and, to a lesser extent, in the cytoplasm of VCT where it forms a connection between neighboring VCTs, and between VCT and overlying SynTB (arrowheads). Snail (stained red) is localized mostly in the nuclei of VCT and mesenchymal cells (short arrows). A normal placenta (A) and a preeclamptic placenta (B) are shown. In preeclampsia, there is a significant reduction in Snail expression. The premature placenta (C) shows a similar staining pattern to the term placenta. The maternal-fetal interface of a preterm placenta (D) reveals expression of Snail in EVT (long arrows), which lack e-cadherin on their membranes. Note that EVTs which express e-cadherin are Snail negative. The loss of e-cadherin by EVT is believed necessary for proper invasion and implantation

Western blotting examination of proteins extracted from human placentas showed that the level of Snail protein was decreased on average by 30% in preeclampsia compared to normal term (Figure 1). Immunohistochemical analysis demonstrated intensive staining of the Snail protein in the villi of normal term placenta where it was localized in the nuclei of two different cell populations, the mesenchymal cells and VCTs. In the decidua, Snail positive nuclei were found in EVTs which were negative for e-cadherin staining (Figure 2). In line with Western blotting data, the preeclamptic placentas show fewer Snail-positive cells. Western blotting revealed variable expression of Snail among the preterm placentas. No statistically significant differences were found between preterm and term placentas or between preterm and preeclamptic placentas after analysis of Snail expression by Western blotting. However, there was a strong trend toward statistical significance between preterm and preeclamptic placentas (Figure 1).

The expression of e-cadherin was lower (by 30%) in term placentas than in preterm and preeclamptic placentas as was demonstrated by Western blotting (Figure 1). However, a statistically significant difference in e-cadherin expression was found between term and preeclamptic samples only. Reduction in e-cadherin protein level in the term placentas compared to preterm ones is in agreement with widely appreciated phenomenon that the relative number of proliferative VCTs is decreased close to the term [2]. Immunohistochemical analysis demonstrated strong staining for e-cadherin in VCTs in both preterm and preeclamptic placentas (Figure 2). E-cadherin connects neighboring VCT-VCT and VCT-SynTB interfaces, and is expressed by proliferative VCTs. E-cadherin is down-regulated during the fusion of more differentiated VCTs to SynTB [31].

**Figure 3:**
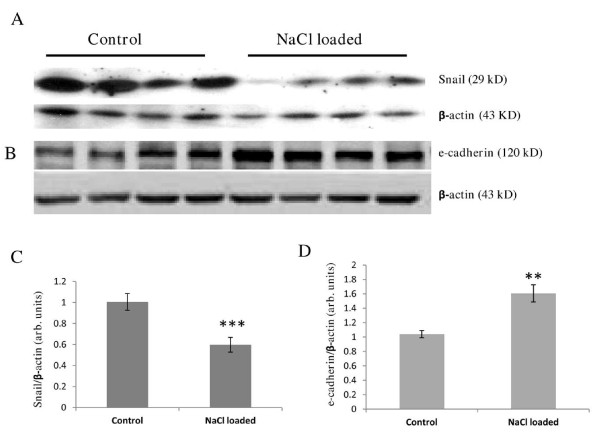
**Western blotting analysis of Snail and e-cadherin expression in the total protein extracts from rat placentas**. Representative immunoblot from 3 independent runs for each sample (Snail) (A), and graph demonstrating densitometry analysis of Snail protein levels in the placentas of control pregnant rats and rats loaded with NaCl (n ≥ 15) (C). Representative immunoblot from 2 independent runs for each sample (e-cadherin) (B), and graph showing densitometry analysis of e-cadherin levels in the two groups studied (n ≥ 10) (D).**p < 0.01, *** p < 0.001

**Figure 4:**
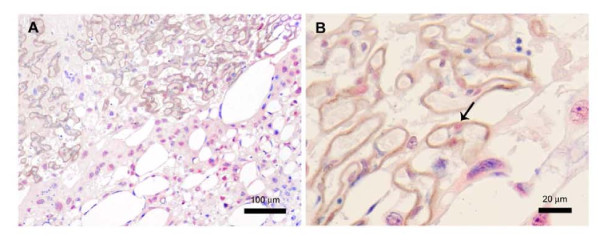
**Immunohistochemistry of Snail and e-cadherin expression in normal definitive rat placenta**. The photomicrographs show that Snail (stained red) is present in the nuclei of spongiotrophoblast cells of the junctional zone. E-cadherin (stained brown) is localized to the SynTB of the labyrinth (A). Snail positivity is also found in mononuclear sinusoidal TGC in some sinusoids positioned close to the junctional zone (arrow) (B)

Pregnant rats receiving high dose of NaCl during the last week of gestation develop symptoms of preeclampsia such as high blood pressure, proteinuria, fetal growth restriction, and enhanced vascular reactivity [24-26,29]. Unrelated to human preeclampsia, additional, pathophysiological changes in pregnant rats used in the study included significantly less weight gain (~7% vs ~36%) and fewer (2-3) pups than in normal pregnancy[24,29]. Western blotting analysis demonstrated that in the placentas of rats with salt-induced preeclampsia, Snail protein expression is decreased by 40% and e-cadherin level increased by 60% compared to control pregnant rats (Figure 3). Immunohistochemical staining of the rat definitive placenta with anti-Snail antibodies revealed strong nuclear and weak cytoplasmic presence of Snail in spongiotrophoblasts in the junctional zone, and in the sinusoidal trophoblast giant cells (TGC) on the periphery of the labyrinth (Figure 4). Immunohistochemical analysis confirmed that in the rat term placenta e-cadherin is present in the SynTB covering the maternal sinusoides of the labyrinth (Figure 4) [32].

## Discussion

In this study, we have demonstrated that human placentas from patients with preeclampsia contain significantly reduced level of the transcription factor Snail compared with normal term placentas. We also showed that in the human placenta, Snail protein is expressed in the VCTs and mesenchymal cells of the villi, and in the EVTs of the decidua. Our data only partially agree with the previous analysis of Snail protein distribution in the human placenta by Blechschmidt et al. [22]. These authors found strong Snail immunoreactivity in the ETVs of the basal plate and weak Snail immunoreactivity in the VCTs using antibodies which are not recommended specifically for immunohistochemistry (Sn9H2, ab31787, Abcam). (We tested this antibody on the human placenta sections and found very weak and irregular immunoreactivity in the deciduas.) Based on calculations of the immunostaining intensity score for e-cadherin and Snail-positive EVTs in the basal plate, Blechschmidt et al. [22] reported an increase in Snail immunoreactivity levels accompanied by a reduction of e-cadherin immunoreactivity in preeclamptic placentas compared to normal term placentas. Yet, our observation does not contradict Blechschmidt et al. for at least two reasons. First, we estimated Snail/e-cadherin levels in total homogenates of large pieces of placenta, where the majority of proteins belongs to villous tissue; and second, there is only limited invasion of EVTs in decidual segments of the placental edges [33]. Thus, any changes in Snail/e-cadherin expression in the EVT of the basal plate in our samples of placenta wouldn't significantly alter total levels of these proteins. Our data for e-cadherin expression in the villous compartment of normal and preeclamptic placentas are in agreement with several reports [11,34,35].

Proper spatio-temporal induction, activation and degradation of Snail may be essential for the control of the VCT maturation and thus the maintenance of continuous, uninterrupted syncytium. In addition to e-cadherin repression, Snail regulates cell cycle progression and confers resistance to cell death as has been previously shown for fetal hepatocytes, epithelial cells, and mouse embryos [36-38]. In preeclampsia, the syncytial layer of the villi in some areas is discontinuous, while in others parts it forms the thick aponecrotic knots and sprouts [4]. Mesenchymal cells of the villi, in contrast to the trophoectoderm-derived epithelial-like VCTs, originate from extraembyonic mesoderm, which is formed during primary EMT at the early stages of human development. Thus, villous mesenchymal cells may retain Snail expression from this period onward. The exact role(s) of Snail in the stroma remains to be elucidated and might be related to the regulation of fibroblast/myofibroblast differentiation and angiogenesis. Remarkably, morphologically preeclamptic placentas are distinctive due to the altered branching pattern of the villous tree, particularly when preeclampsia is combined with IUGR [2,4]. Thus, down-regulation of Snail by internal and external factors may contribute to the development of human preeclampsia by aberration of essential stages of placental development.

In the rat definitive placenta, Snail is highly expressed in spongiotrophoblasts, the TGC precursor cells in the junctional zone (Figure 4). We also found focal expression of Snail in the sinusoidal TGC at the periphery of the labyrinth. Rodent and human placentas are similar in basic morphology and functions. However, there are some differences in the structure and formation of the chorion [39]. Rodent labyrinth, which is functionally analogous to primate chorionic villi, is lined from the maternal side with one layer of mononuclear sinusoidal TGC and two layers of SynTB. The SynTB contacting fetal mesenchyme originates from chorionic ectoderm enriched with e-cadherin positive progenitor cells. The outer SynTB and sinusoidal TGC are derived from the inner part of the ectoplacental cone where the majority of trophoblast progenitor cells, committed to SynTB fate, persistently express e-cadherin. Before 8dpc of rat placental development, e-cadherin is localized to trophoblasts; at 9dpc its expression is confined exclusively in trophoblast derived cells at the extraembyonic ectoderm, and from 10dpc onward, e-cadherin remains exclusively expressed in the labyrinth [32]. Thus, in the outer layers of the ectoplacental cone, e-cadherin is lost from 7dpc onward when most of the Snail-positive cells appear. *In situ *hybridization analysis demonstrated that from 7.5dpc Snail transcripts were detected in the ectoplacental cone in precursor cells of TGC and, at much lower levels, in the proliferative trophoblasts of the chorion [40,41]. At 10.5dpc Snail transcripts were detected in the spongiotrophoblast layer and in some focal areas of the labyrinth [41]. Therefore, only a small number of Snail-positive cells are present in the developing labyrinth before 13dpc. The junctional zone encloses a highly proliferative cell population until day 14 of gestation, afterward the mitotic activity at this zone ceases sharply [42]. Thus, an up-regulation of Snail in the junctional zone spongiotrophoblasts may inhibit their proliferation [36]. Notably, down-regulation of Snail expression takes place at the border between the population of spongiotrophoblasts and endocycling TGC [20]. In Rho-1 trophoblast cell line, down-regulation of Snail promotes entry into endoreduplication through control of the "G2 decision point" [20]. The trophoblasts are the only type of mammalian cells that posses the ability to quit mitosis and enter into endocycle, thus multiplying DNA content in the range 8 N-64 N in the human and up to 4096 N in rodents [42-44]. These polyploid cells form an invasive front when they contact maternal decidua and play a crucial role in placental development and its communication with maternal tissues [42-44].

In rodents, in contrast to humans, e-cadherin and Snail are persistently expressed in the majority of cells positioned in two different compartments throughout placental development. The only exception is a small number of mononuclear TGC in the sinusoids located close to the junctional zone (Figure 4). Interestingly, SynTB progenitor cells in rodent placenta (like VCT in human) have never been verified [39].

We have also found that Snail is down-regulated and e-cadherin is up-regulated in rat placentas, when pregnant rats developed preeclampsia-like symptoms in response to NaCl overload. How can the expression of these proteins be coupled if they are expressed by distinct populations of cells? We speculate that decreased levels of Snail in the spongiotrophoblasts of salt-loaded pregnant rats could boost the endoreduplication process, thus amplifying the number of TGC. The TGCs produce activin, a TGFβ family member protein, which can trigger differentiation of progenitor trophoblast cells away from spongiotrophoblast/TGC fate toward SynTB as suggested in Natalie et al. [45]. Therefore, in the rat, Snail functions appear to be restricted to the control of spongiotrophoblast differentiation.

Snail expression, stability and activity are under control of integrated and complex cellular signaling networks which can be furthermore affected by perturbations in the oxygen level [14,46]. Oxidative stress triggers Snail transcription directly through elevation of ROS or by activation of NF-κB signaling pathway [14,47]. From the other side, hypoxia promotes Snail protein stability by inhibiting Snail proteasome degradation. HIF-1α has been shown to down regulate F-box E3 ubiquitin ligase FBXL14, which targets specifically the cytoplasmic portion of Snail in some cancerous cell lines [17]. Additionally, two highly HIF-up regulated proteins, lysyl oxidase (LOX) and LOX-like 2 (LOXL2) can stabilize Snail and therefore may alter its activity, however, this interaction seems to be cell-line specific [48,49]. Interestingly, while hypoxia strongly elevates LOX gene expression, its enzymatic activity requires subsequent re-oxygenation [50]. LOX and LOXL2, which are generally known as classical proteins responsible for the cross linking of collagen and elastin, are normally expressed in the human placenta, and in the VCT and SynTB particularly, throughout gestation [51]. Since preeclamptic placentas are characterized by increased oxidative stress [52], over-expression of active HIF-1α and 2-α [53] and reduced activity of proteasomal proteins [54], an up-regulation of Snail in human preeclampsia might be suggested. However, the opposite--the reduction of Snail protein--may indicate that other factors associated with pregnancy and/or preeclampsia may have a greater effect on Snail, and thus affect normal placental development. Indeed, compromised oxygenation of placentas in preeclampsia is thought to be secondary to the primary cause of preeclampsia--placental insufficiency due to defective trophoblast functions [3,6,52,55].

The similarity between Snail down-regulation in human preeclampsia and in the rat model may provide additional information about factors which may modulate Snail expression and stability in placentas. The last week of normal rat pregnancy is characterized by expansion of the labyrinth, cessation of spongiotrophoblast proliferation, and TGC invasion of the uterine mesometrial compartment [27]. These developmental events coincide with a significant decrease in maternal blood pressure. Maternal insult in the form of a 20-fold increase in dietary salt during this period of gestation causes a dramatic elevation of blood pressure accompanied by increased placental oxidative stress and inflammation [25].

Throughout normal pregnancy, maternal blood pressure is decreased despite dramatic increases in extracellular fluid volume and salt retention due to persistent activation of the renin-angiotensin-aldosterone system (RAAS) and placental secretion of the endogenous cardiotonic steroids, inhibitors of Na, K-ATPase [56,57]. RAAS, especially its utero-placental component, has been recently implicated in the pathology of preeclampsia [56]. Particularly, angiotensin II and IV have been shown to regulate invasion, migration and apoptosis of the rat and human trophoblasts [56,58]. These facts suggest that utero-placental and circulating hormones involved in blood pressure control during pregnancy might play a more important role in the direct regulation of trophoblast differentiation than has been appreciated.

Although no significant correlations were found between salt consumption and preeclampsia development, some epidemiological studies reported small yet significant correlation between salt intake and hypertension in humans [59]. Recent data demonstrated higher salt sensitivity and pressor response to salt loading in women with a history of hypertensive pregnancy when compared to normotensives [60]. Placental and maternal changes in the salt-induced rat model of preeclampsia mirror many of those noted in humans [25,26,61,62]. However, unlike in humans, rats with preeclampsia symptoms due to salt-overload demonstrated large (~44%) decrease in food intake, significant reduction of pregnancy-related maternal weight gain and fewer pups [24,26]. Caloric restriction of 50% during the second half of rat pregnancy alone can cause a decrease of maternal and fetal weight and placental alterations [63]. Placenta, and labyrinth specifically, from undernourished pregnant rats have significantly increased apoptosis [64]. However, apoptosis is elevated in a variety of pregnancy complications including preeclampsia [65]. An increased rate of placental apoptosis was shown in the salt-induced rat model as well [25]. Since there are no reports demonstrating caloric restriction as a possible risk factor for preeclampsia development, maternal undernutrition in pregnant rats on a high sodium uptake is one of the limitations of this model and should be considered in the design of future studies.

## Conclusions

This study demonstrates that in the human and rat placentas Snail is expressed in trophoblast and mesenchymal progenitor cell lineages during their transition to the terminally differentiated stage. These observations are congruent with previously reported Snail expression in some of these lineages, such as rat spongiotrophoblasts, human EVT and trophoblast cell lines. We also found that in human preeclampsia and in the rat salt-induced model of preeclampsia, the level of transcription factor Snail is significantly decreased in total placenta extracts. The reduced levels of Snail might trigger alterations in diverse pathways of placental cell lineage differentiation and thus contribute to the development of preeclampsia and/or IUGR.

## Competing interests

The authors declare that they have no competing interests.

## Authors' contributions

LF participated in the study design, carried out experiments and prepared the manuscript. CGW performed histopathological and immunohistochemical analysis and contributed to the manuscript preparation. SS and NK collected the human placenta samples, extracted placental proteins and participated in Western blotting analysis. JIS and TH formulated the research questions, conceived the study and participated in drafting of the manuscript. DM participated in the development of critical methodology and the manuscript preparation. All authors read and approved the final manuscript.
